# Genome-Wide Identification of *BrCAX* Genes and Functional Analysis of *BrCAX1* Involved in Ca^2+^ Transport and Ca^2+^ Deficiency-Induced Tip-Burn in Chinese Cabbage (*Brassica rapa* L. ssp. *pekinensis*)

**DOI:** 10.3390/genes14091810

**Published:** 2023-09-17

**Authors:** Shuning Cui, Hong Liu, Yong Wu, Lugang Zhang, Shanshan Nie

**Affiliations:** State Key Laboratory of Crop Stress Biology for Arid Area, College of Horticulture, Northwest A&F University, Xianyang 712100, China; cuishuning322@163.com (S.C.); lh990812999@163.com (H.L.); wuyongfjiur@163.com (Y.W.); lugangzh@163.com (L.Z.)

**Keywords:** Chinese cabbage, *BrCAX* genes, Ca^2+^ deficiency stress, tip-burn, expression analysis

## Abstract

Calcium (Ca^2+^) plays essential roles in plant growth and development. Ca^2+^ deficiency causes a physiological disorder of tip-burn in Brassiceae crops and is involved in the regulation of cellular Ca^2+^ homeostasis. Although the functions of Ca^2+^/H^+^ exchanger antiporters (CAXs) in mediating transmembrane transport of Ca^2+^ have been extensively characterized in multiple plant species, the potential roles of *BrCAX* genes remain unclear in Chinese cabbage. In this study, eight genes of the *BrCAX* family were genome-widely identified in Chinese cabbage. These BrCAX proteins contained conserved Na_Ca_ex domain and belonged to five members of the *CAX* family. Molecular evolutionary analysis and sequence alignment revealed the evolutionary conservation of *BrCAX* family genes. Expression profiling demonstrated that eight *BrCAX* genes exhibited differential expression in different tissues and under heat stress. Furthermore, Ca^2+^ deficiency treatment induced the typical symptoms of tip-burn in Chinese cabbage seedlings and a significant decrease in total Ca^2+^ content in both roots and leaves. The expression changes in *BrCAX* genes were related to the response to Ca^2+^ deficiency-induced tip-burn of Chinese cabbage. Specially, *BrCAX1-1* and *BrCAX1-2* genes were highly expressed gene members of the *BrCAX* family in the leaves and were significantly differentially expressed under Ca^2+^ deficiency stress. Moreover, overexpression of *BrCAX1-1* and *BrCAX1-2* genes in yeast and Chinese cabbage cotyledons exhibited a higher Ca^2+^ tolerance, indicating the Ca^2+^ transport capacity of *BrCAX1-1* and *BrCAX1-2*. In addition, suppression expression of *BrCAX1-1* and *BrCAX1-2* genes reduced cytosolic Ca^2+^ levels in the root tips of Chinese cabbage. These results provide references for functional studies of *BrCAX* genes and to investigate the regulatory mechanisms underlying Ca^2+^ deficiency disorder in Brassiceae vegetables.

## 1. Introduction

Calcium (Ca^2+^) is an essential plant macro-nutrient and plays crucial roles in strengthening the cell wall and membrane and maintaining cell integrity [1]. Ca^2+^ functions as a second messenger in multiple signaling cascades that regulate plant growth and development and respond to biotic and abiotic stresses [2,3,4]. Intercellular Ca^2+^ levels are tightly controlled by Ca^2+^ storage and transport system, which modulates the responses to various environmental stimuli [5,6]. The involvement of Ca^2+^ transport in eliciting the fluctuations of cytosolic Ca^2+^ is attributed to the activities of three major classes of membrane transporters, including Ca^2+^ channels, Ca^2+^-ATPases (pumps), and Ca^2+^/cation antiporters [7,8]. The coordination of these Ca^2+^ transporters mediate the movement of Ca^2+^ across diverse biological membranes, such as plasma membrane (PM), tonoplast (TN), and endoplasmic reticulum (ER) membrane, which contributes to a dynamic balance of cytosolic Ca^2+^ that depends on influx and efflux of Ca^2+^ [9].

Ca^2+^/H^+^ exchanger antiporters (CAXs), belonging to one class of transporter proteins of Ca^2+^/cation antiporter (CaCA) superfamily, perform housekeeping functions to restrict the accumulation of excessive cations and to transport them out of the cytosol across membranes [10,11]. As ion-coupled antiporters, CAXs are predominately localized in TN and mediate the transport of Ca^2+^ across TN against the concentration gradient of Ca^2+^ [11,12]. CAX family proteins have been reported to extensively exist in bacteria and higher plants [11,13]. The innate function of CAX is credited to the structure of membrane helices, which is characterized by highly conserved 11 transmembrane (TM) regions [14]. The important sequence diversities in the loop and tail regions of N- and C-terminal among different CAXs indicate the activity of CAXs having a wide range of cation specificity [14]. Plant CAXs have been functionally characterized across multiple species and are classified into two distinct subgroups of I-A and I-B types based on the phylogenetic analysis of CAX family proteins [15,16].

The CAX proteins from *Arabidopsis thaliana* are most thoroughly studied in biological functions, such as ion transport, abiotic stress signaling, and Ca^2+^ homeostasis [5,17]. In addition to the evidence of transport properties of CAX using yeast heterologous expression, ectopic expression and knockout mutant analyses sufficiently show the functions of CAX in planta [15,18]. Several analyses of *Arabidopsis cax* mutants indicate the phenotypes of an increased sensitivity to ion toxicity and abiotic stress [19,20,21]. *Arabidopsis* CAX1 and CAX2 transporters have been demonstrated the regulatory roles in cation transport [22,23]. A *cax4* loss-of-function mutant suggests the important modulation of *CAX4* gene in root growth of *Arabidopsis* under heavy metal stress conditions [24]. The *cax1/cax3* double mutation exhibits necrosis of the leaf tips and shoot apex in *Arabidopsis* and suggests the exchange of cytosolic Ca^2+^ by CAX1 and CAX3 antiporters [20,25]. Overexpression of the N-terminal truncated *Arabidopsis CAX1* in tobacco increases Ca^2+^ accumulation in leaves and roots and improves salt stress sensitivity [26,27]. Remarkably, although the line of deregulated *CAX1* exhibits a higher Ca^2+^ concentration in tissues, it shows an evident increase in the symptoms of Ca^2+^ disorders [20,28]. In tomato plants, the high expression of deregulated *CAX1* presents the phenotype of Ca^2+^ disorder in fruit, such as a higher accumulation of total Ca^2+^ and the increased incidence of fruit necrotic lesions [29,30]. The inconsistent correlation between total tissue Ca^2+^ and incidence of Ca^2+^ disorder may be the result of abnormal cellular Ca^2+^ partitioning and distribution [30].

Ca^2+^ deficiency disorder is a physiological disorder, such as black heart in celery [31], blossom-end rot (BER) in tomato [32], bitter pit in apple [33], and tip-burn in Brassiceae leafy vegetables [34,35], and directly affects horticulture crop production causing significant economic losses [1,3,36]. Considerable studies have suggested the two primary causes of Ca^2+^ deficiency disorder, including localized Ca^2+^ deficiency and aberrant Ca^2+^ homeostasis, which are linked to cellular Ca^2+^ transcript and CAX activity [15,36]. Chinese cabbage (*Brassica rapa* L. ssp. *pekinensis*), belonging to Brassicaceae family, is an economically important leafy vegetable grown worldwide. Ca^2+^ deficiency-induced tip-burn mainly occurs in the inner fast-grown leaves of Chinese cabbage, leading to a serious threat to quality and yield [34]. However, the underlying mechanism of cellular Ca^2+^ transmembrane transport and tip-burn incidence remains largely unveiled in Chinese cabbage. In this study, to understand the specific expression and putative function of *CAX* genes in response to environmental stresses and Ca^2+^ deficiency conditions, eight genes from *BrCAX* family were genome-widely identified and characterized in Chinese cabbage. The temporal and spatial expression profiles of *BrCAX* genes were analyzed under different Ca^2+^ deficiency conditions. Moreover, the Ca^2+^ transport capacities of *BrCAX1-1* and *BrCAX1-2* genes were validated by overexpression in yeast and Chinese cabbage cotyledons and transient suppression expression in Chinese cabbage. The results of this study will provide references for functional studies of *BrCAXs* in regulating Ca^2+^ transport and Ca^2+^ deficiency-induced tip-burn in Chinese cabbage and other vegetable crops. 

## 2. Materials and Methods

### 2.1. Plant Materials and Treatments

The Chinese cabbage tip-burn susceptible line ‘10S42′ and resistant line ‘10S230′ were used in this study. Germinated seeds were grown in 1/4 Hoagland nutrient solution under standard growth conditions with 7 d. Subsequently, the seedlings were transferred to different treatments with 4, 1.5, and 0.75 mM Ca^2+^ contents, keeping the consistent content of other elements, especially N, P, and K. The normal Hoagland contained 4 mM Ca^2+^ contents. The conditions with 1.5 and 0.75 mM Ca^2+^ contents were considered Ca^2+^ deficiency treatments. For gene expression analysis, the tissues, including root, leaf base, and leaf apex, were collected at 28 d after treatment. The symptom of tip-burn was observed every 7 d under treatment. For the determination of total Ca^2+^ content, the roots and leaves of seedlings were also collected at 28 d after treatment.

### 2.2. Genome-Wide Sequence Searching and Identification of BrCAX Family Genes

The known sequences of CAX family proteins from *A. thaliana* were downloaded from the Arabidopsis Information Resource (TAIR) database and used as BLASTP query to search against the Brassicaceae database (BRAD). On the other hand, Hidden Markov Model (HMM) file of Na_Ca_ex domain (PF01699) was downloaded from Pfam database and subjected to search against the genomic sequences of Chinese cabbage using HMMER (version 3.2.1) search tool with an E-value cut-off of 1.0. After removing the repeat sequences from the two searching results, candidate sequences were verified by analyzing conserved Na_Ca_ex domain through public databases, including NCBI Conserved Domain Database (http://www.ncbi.nlm.nih.gov/cdd, accessed on 20 February 2023), Pfam (http://pfam.xfam.org, accessed on 20 February 2023), and SMART (http://smart.embl-heidelberg.de, accessed on 20 February 2023). The obtained proteins were named as BrCAXs based on their homologous annotations. The physiological and biochemical characteristics of BrCAX proteins were analyzed using ProtParam tool of ExPASy (http://web.expasy.org/protparam, accessed on 22 February 2023). Subcellular localization of BrCAX proteins was predicted by online WoLF PSORT program (https://wolfpsort.hgc.jp/, accessed on 22 February 2023). The exon–intron structures of *BrCAX* genes were analyzed using the online Gene Structure Display Server (GSDS 2.0; http://gsds.cbi.pku.edu.cn/, accessed on 22 February 2023) with default parameters.

### 2.3. Phylogenetic Analysis and Syntenic Analysis of BrCAX Genes

The genome-wide searches of *CAX* family genes from other five *Brassica* species, including *Brassica napus*, *Brassica oleracea*, *A. thaliana*, *Arabidopsis lyrata*, and *Raphanus sativus*, were performed to obtain the CAX homologous protein sequences from BRAD database (Table S1). Multiple sequence alignment was performed using DNAMAN 9.0 software. Phylogenetic analysis was performed using MEGA 11.0 software with neighbor-joining (NJ) method and bootstrap values of 1000 replicates. Chromosomal localization and syntenic analysis were performed using TBtools 1.105 software based on *B. rapa* chromosome information in BRAD database.

### 2.4. Positive Selection Analysis of BrCAX Genes

The *Ka* (nonsynonymous substitution rate) and *Ks* (synonymous substitution rate) were calculated to detect positive selection of CAX homologs. The protein sequences of CAX homologs from six *Brassica* species were aligned using ClustalX 2.1 software. Alignment results were submitted to PAL2NAL (http://www.bork.embl.de/pal2nal, accessed on 15 March 2023) and translated into coding sequence alignments. The output alignments were used to estimate the values of *Ka*, *Ks*, and *Ka*/*Ks* ratio using KaKs Calculator 2.0 software with Model Selection (MS) method. The boxplot of pairwise comparisons among CAX homologs was constructed using R program.

### 2.5. Prediction of BrCAX Protein Structures

The three-dimensional (3D) protein structures of BrCAXs were predicted using the homology model method by Phyre2 web [37]. The known template of c4k1c from *Saccharomyces cerevisiae* VCX1 (NCBI: CAA98696.1) protein was used for homology modeling of BrCAX proteins. The obtained 3D structures of BrCAX proteins were visualized using PyMOL Viewer 2.5 software. Structural alignment and amino acid residue analysis were also performed.

### 2.6. Prediction of Cis-Acting Elements in BrCAX Promoters

The promoter sequences (the 2.0 kb upstream sequences of the 5′ regulatory regions) of *BrCAX* genes were retrieved from BRAD database. The putative cis-acting elements were predicted using PlantCARE program (http://bioinformatics.psb.ugent.be/webtools/plantcare/html/, accessed on 15 March 2023) and visualized using TBtools 1.105 software.

### 2.7. Expression Analysis of BrCAX Genes

The tissue-specific expression patterns of *BrCAX* genes were analyzed using the available RNA-Seq data of Chinese cabbage ‘Chiifu’ [38]. The FPKM values from root, stem, leaf, flower, silique, and callus were downloaded. Gene expression pattern was analyzed using the values of log_2_ FPKM. In addition, the probe intensity (PI) value of *BrCAX* gene expressions under 45 °C heat stress was obtained from a previous microarray analysis on Chinese cabbage ‘Chiifu’ [39]. Expression pattern of *BrCAX* genes was analyzed using the values of log_2_ fold change (PI in heat stress/PI in control). Heat map of gene expression was drawn using TBtools 1.105 software.

Further expression analysis of *BrCAX* genes in response to Ca^2+^ deficiency stress was performed by quantitative real-time PCR (qRT-PCR). Total RNA extraction and cDNA synthesis were performed according to the previous reports [40]. The qRT-PCR analysis was conducted with three biological replicates using Master qPCR Mix (SYBR Green I; TsingKe Biotech Co., Ltd., Beijing, China) on an iCycler iQ5 Real-Time PCR Detection System (Bio-Rad, Hercules, CA, USA). The specific primer sequences were designed using Beacon Designer 7.0 software (Table S2). The *EF-1α* gene (Bra031602) of *B. rapa* was used as internal reference gene to normalize expression data. The relative expression was calculated using delta-delta-CT method.

### 2.8. Protein Interaction Network

An interaction network of BrCAX proteins was predicted using STRING (http://stringdb.org, accessed on 15 March 2023) with the parameter of the minimum required interaction score ≥ 0.7. The interaction relationships were analyzed according to the genome information of Chinese cabbage from BRAD and NCBI databases. The predicted interacting proteins were annotated based on KEGG and NCBI databases.

### 2.9. Transformation of BrCAX1-1 and BrCAX1-2 Genes in Yeast Mutant

The full-length CDS sequences of *BrCAX1-1* and *BrCAX1-2* genes were cloned from the leaves of Chinese cabbage ‘10S42′. According to the known specific N-terminal autoinhibitory region of AtCAX1 from *A. thaliana* [41], the N-terminal truncated sequences of *BrCAX1-1* (−37 aa) and *BrCAX1-2* (−36 aa) (labeled *BrCAX1-1-N* and *BrCAX1-2-N*) were also obtained from the full-length sequences. The obtained sequences were inserted into the yeast expression vector pDR196 and transferred into the Ca^2+^ sensitive yeast mutant strain k667 (*cnb1::LEU2 pmc1::TRP1 vcx1*∆) [42], according to the reported method [43]. The used primers are presented in Table S2. The yeast strain W303 and the yeast expressing empty vector pDR196 were separately used as the positive and negative controls. For Ca^2+^ tolerance assay of yeast, the prepared yeast suspensions (OD_600_ = 1.0) were serially diluted (10^−1^, 10^−2^, 10^−3^, and 10^−4^) and then spotted onto YPDA/SD-ura medium containing different concentrations of CaCl_2_ (0, 5, 25, 50, 100, and 200 mM). Meanwhile, the yeast solutions cultured to the exponential phase were transferred to liquid YPDA/SD-ura medium containing 50 mM CaCl_2_ and further cultured. The OD values were measured every 2 h and then used to construct the growth curves of different yeast strains. The yeast cells from different transformants under 25 mM CaCl_2_ treatment were also collected for measuring the total Ca^2+^ content. For Ca^2+^ fluorescence staining assay, the yeast cells (OD_600_ = 0.4~0.5) under 50 mM CaCl_2_ treatment were washed using PBS solution three times and incubated with 15 μM Fluo4-AM for 1 h at 30 °C. A confocal laser scanning microscope (LEICA TCS SP8, Wetzlar, Germany) was used to observe the green fluorescence signaling under the excitation and emission wavelengths with 488 nm and 512~520 nm. The ImageJ 1.51 software was used to determine the grey values of green fluorescence in yeast cells.

### 2.10. Infiltration and Transformation of Chinese Cabbage Cotyledons

The *BrCAX1-1-N* and *BrCAX1-2-N* sequences were inserted into the pCAMBIA-2300 vector containing green fluorescent protein (GFP) sequence and transferred into *Agrobacterium* strain GV3101. The infiltration experiments of Chinese cabbage cotyledons were performed according to the previously reported methods with minor modifications [35,44]. The *Agrobacterium* strain containing the target sequences with 1 mL were infiltrated into the back of cotyledons of Chinese cabbage seedlings (5 d old). After 24 h of infiltrating, the seedlings were sprayed with 50 mM CaCl_2_ solutions per 12 h and cultured in the dark with 2 d. The cotyledons infiltrated with the empty vector strain and sprayed with equivalent ddH_2_O were used as the controls. Observation of GFP fluorescence was performed using the confocal laser scanning microscope (LEICA TCS SP8, Wetzlar, Germany) and used to validate the successful infiltrating. The infiltrated cotyledons were collected for gene expression analysis as the above described. The phenotype and disease index of cotyledon necrosis were determined as follows: “0” representing no obvious necrosis, “1” representing the necrosis area < 50% of cotyledon area, and “2” representing the necrosis area > 50% of cotyledon area. The disease index was calculated using 30 seedlings per replicate, with three independent replicates.

### 2.11. Anti-Sense Oligonucleotides (AS-ODN) Treatment

Gene suppression expressions of *BrCAX1-1* and *BrCAX1-2* in Chinese cabbage were performed using anti-sense oligonucleotides (AS-ODN) treatment as described previously with minor modifications [45]. The 7 d seedlings from Chinese cabbage ‘10S42′ were used in this assay. The specific AS-ODN and S-ODN sequences were designed using Soligo (http://sfold.wadsworth.org/cgi-bin/soligo.pl, accessed on 5 May 2023) (Table S2). The seedlings were put in 1.5 mL tubes containing 1 mL 1/4 Hoagland nutrient solution and 20 μM ODN. After 2 d of treatment, the seedlings were collected for gene expression validation. Ca^2+^ fluorescence of the root tips was observed using the confocal laser scanning microscope (LEICA TCS SP8, Wetzlar, Germany) after incubating with Fluo4-AM, according to the reported method by Li et al. [46].

### 2.12. Total Ca^2+^ Content Measurement

The samples for the measurement of total Ca^2+^ contents were collected, washed, and dried at 85 °C for 24 h. Then, the samples with 0.1 g dry weight were dissolved in 6 mL HNO_3_. An atomic absorption spectrophotometer (ZA3000, HITACHI, Tokyo, Japan) was used to detect the total Ca^2+^ content according to the method by Bai et al. [47].

### 2.13. Statistical Analysis

One-way ANOVA and Duncan’s post hoc tests in SPSS 24.0 software were used to analyze the data. Statistical significance was defined as *p* < 0.05. The data were presented as the means ± SEM from at least three independent experiments. 

## 3. Results

### 3.1. Identification and Characterization of BrCAX Genes in Chinese Cabbage

**Figure 1:**
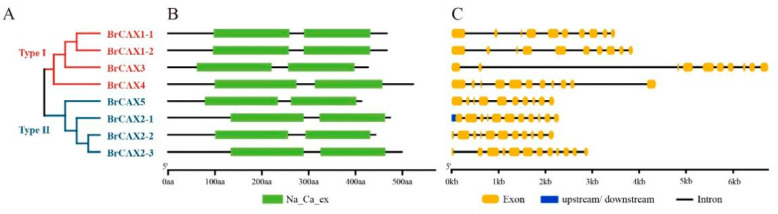
Characterization of *BrCAX* family genes in Chinese cabbage. (**A**) Phylogenetic analysis and classification of eight *BrCAX* genes. (**B**) The conserved domains of *BrCAX* genes. (**C**) The distribution of exon–intron structure of *BrCAX* genes.

In this study, to identify *BrCAX* family genes in Chinese cabbage, the gene sequences of *Arabidopsis CAX* family were used to perform the genome-wide BLASTP and HMM search against BRAD database. As a result, a total of eight candidate sequences belonging to five genes were obtained and considered as *BrCAX* family members in Chinese cabbage (Table 1). Based on the information of BRAD, coding sequences (CDS) and amino acid sequences of *BrCAX* family genes were retrieved and used for further analysis. The length of *BrCAX* genes ranged from 1236 to 1494 nucleotides, coding 411 to 497 amino acids (Table 1; Table S3). Analysis of the phylogenetic relationship suggested that these BrCAX proteins were classified into two distinct subgroups: Type I and Type II (Figure 1A). Verification of conserved domains indicated that these BrCAXs contained typical protein structures that were characterized by conserved Na_Ca_ex domains (Figure 1B), supporting the credibility of genome-wide searching. Further subcellular localization for all the BrCAX proteins was predicted in the vacuole (Table 1). Gene structure analysis showed that several *BrCAX* genes from the same subgroup had similar exon–intron composition patterns (Figure 1C), such as *BrCAX1-1*, *BrCAX1-2*, and *BrCAX3* genes that contained the same number of exons and introns. In addition, physiological and biochemical properties of BrCAX proteins were analyzed and are presented in Table S3.

### 3.2. Phylogenetic Analysis and Syntenic Analysis of BrCAX Genes

**Figure 2:**
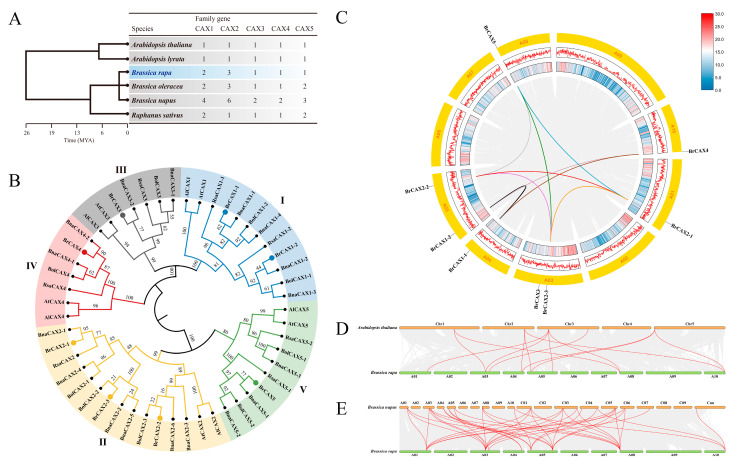
Overview of *CAX* family genes in different *Brassica* species. (**A**) The number of *CAX* family genes from Chinese cabbage and other five *Brassica* species. MYA: million years ago, indicates the evolutionary timescale of species. (**B**) Phylogenetic tree of *CAX* family proteins from Chinese cabbage and other five *Brassica* species. Br: *B. rapa*; Bna: *B. napus*; Bol: *B. oleracea*; At: *A. thaliana*; Al: *A. lyrata*; Rsa: *R. sativus*. (**C**) The localization of eight *BrCAX* genes in the genome of Chinese cabbage. Different color lines indicate the segmental duplication genes. (**D**,**E**) Syntenic analysis of *BrCAX* genes with *CAX* homologs from *A. thaliana* and *B. napus*.

To further explore the phylogenetic relationships of *CAX* homologs, the *CAX* family genes from other five *Brassica* species were identified and used to perform a detailed phylogenetic analysis (Figure 2A). Comparative analysis of *CAX* members showed that the number of *BrCAX* family genes in Chinese cabbage was more than that in the model plant *A. thaliana*. The greatest number of *CAX* family members was in *B. napus*. Phylogenetic analysis showed that these *CAX* homologous genes were obviously classified into five groups, which was coincident with the five members of *CAX* family (Figure 2B). Except the *BrCAX2-2* and *BrCAX2-3* genes, all the *BrCAX* genes showed closer distance to *BnaCAX* genes from *B. napus*, which indicates the similar molecular characterizations between *BrCAX* and *BnaCAX* genes.

To detect the chromosomal distribution of *BrCAX* family genes, these genes were mapped onto ten chromosomes of Chinese cabbage based on the BRAD database. The eight *BrCAX* sequences were distributed across six chromosomes of *B. rapa* (Figure 2C), while no gene was found on the A02, A06, A07, and A09 chromosomes. Two pairs of *BrCAX* genes, including *BrCAX1-1*/*BrCAX1-2* and *BrCAX2-1*/*BrCAX2-2*/*BrCAX2-3*, were duplicated genes and arranged on different chromosomes. Moreover, nine pairs of *BrCAX* genes were identified as segmental duplication genes (Figure 2C), suggesting that these genes undergone segmental duplication events across the genome of Chinese cabbage. Further syntenic analysis indicated that all the *BrCAX* genes were in correspondence to their homologous genes in *A. thaliana* and *B. napus* (Figure 2D,E), which proved the expansion of *CAX* gene family during the evolution of species.

### 3.3. Evolutionary Conservation of BrCAX Genes

**Figure 3:**
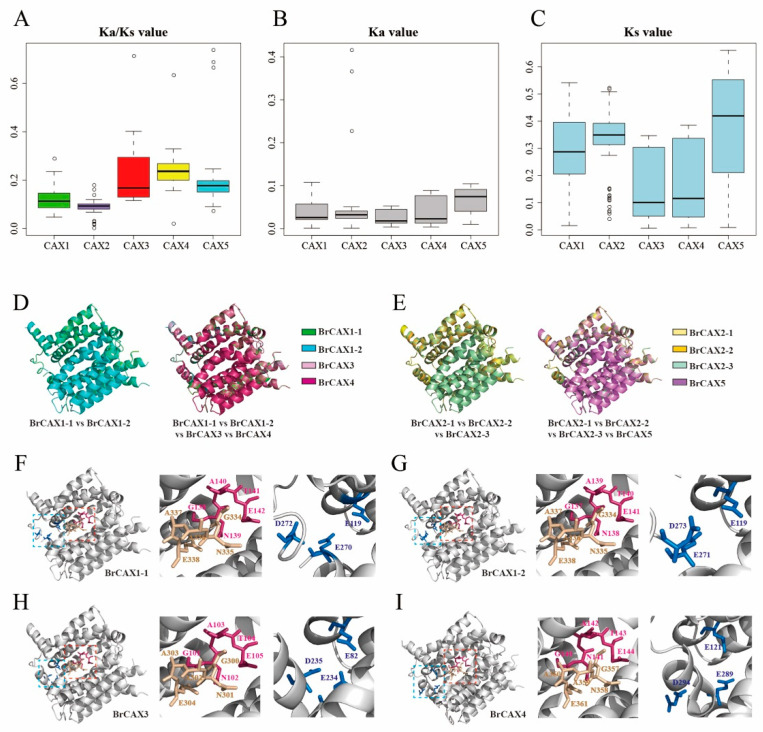
Analysis of molecular evolution of *BrCAX* family genes. (**A**–**C**) The pairwise values of *Ka*/*Ks* ratio, *Ka*, and *Ks* among *CAX* homologous genes from six *Brassica* species. (**D**,**E**) Alignment of the predicted 3D structure of BrCAX proteins. (**F**–**I**) The conserved “GNxxE” regions (in pink and wheat) and the predicted Ca^2+^ binding sites (in blue) in BrCAX1-1, BrCAX1-2, BrCAX3, and BrCAX4 proteins.

The homologous genes of *CAX* family from six *Brassica* species were further used to assess the genetic evolution of *CAX* family genes. Pairwise comparison of *CAX* homologs was performed to calculate the value of *Ka*/*Ks* ratio (Figure 3A–C), which indicates the evolution rates and selective pressure. In general, *Ka*/*Ks* value > 1 indicates positive selection, while *Ka*/*Ks* value < 1 indicates purifying selection. The average *Ka*/*Ks* values of these *CAX* homologs were less than 1 (Figure 3A), representing a significantly purifying selection on *CAX* family genes. Among the five groups of *CAX* homologs, the *Ka*/*Ks* values displayed the order: *CAX4* > *CAX3* > *CAX5* > *CAX1* > *CAX2*. The pairs of *CAX3* and *CAX4* genes showed higher *Ka*/*Ks* values. The pairs of *CAX1* and *CAX2* genes showed lower *Ka*/*Ks* values, which indicated that these genes were more conserved than other *CAX* genes and suggested the more ancient homology of *CAX1* and *CAX2* genes. Moreover, the *Ks* values that represent divergence time also showed higher pairwise values in *CAX1* and *CAX2* genes than that in *CAX3* and *CAX4* genes (Figure 3C). These data indicate the conserved evolutionary pattern of *CAX* family genes among different *Brassica* species.

Furthermore, the 3D structures of BrCAX proteins were predicted using homology modeling against ScVCX1 protein. All the BrCAX proteins represented high similarity to ScVCX1 and had conserved crystal structures with 11 TM helices (Figure S1). Alignment of protein crystal structure was further performed to detect the evolutionary homology among BrCAX proteins (Figure 3D,E). The results suggested that eight BrCAX proteins showed highly similar structures. In particular, the structures of BrCAX1-1, BrCAX1-2, BrCAX3, and BrCAX4 were slightly different only in several residues (Figure 3D). The conserved *α*-repeat regions (GNxxE) of BrCAX proteins were also shown in their crystal structures (Figure 3F–I; Figure S1). Moreover, the known Ca^2+^ binding sites were identified in the structures of BrCAX1-1, BrCAX1-2, BrCAX3, and BrCAX4 proteins (Figure 3F–I), suggesting that these proteins may evolve the conserved functions in Ca^2+^ transport and maintaining Ca^2+^ concentration. These results of molecular evolutionary analysis support the evolutionary conservation of *BrCAX* family genes.

### 3.4. Sequence Alignment of BrCAX Proteins

**Figure 4:**
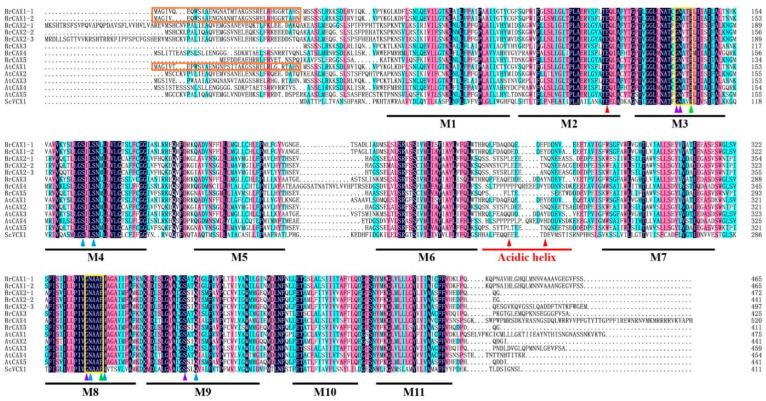
Sequence alignments of BrCAX proteins to the homologous proteins. The transmembrane regions (M1–M11) are marked in black lines. The N-terminal autoinhibitory region is marked in the orange box. The acidic helix region is marked by a red line. The yellow boxes indicate the conserved “GNxxE” motifs. The arrows in different colors indicate the conserved residues.

Alignment of amino acid sequences showed that eight BrCAX proteins shared high similarity to AtCAX and ScVCX1 protein sequences and may have conserved protein functions (Figure 4). Remarkably, similar to AtCAXs and ScVCX1, BrCAX proteins contained the conserved 11 TM regions that were divided into three components: M1, M2-6, and M7-11. The M1 region was considered as a dispensable element for cation transport function [15], and the M2-6 and M7-11 regions were linked by an un-conserved acidic helix region. Furthermore, two highly conserved *α*-repeat regions (the known cation-binding regions) containing “GNxxE” motifs were also identified in M3 and M8 regions. The specific N-terminal autoinhibitory region was identified in BrCAX1-1 (37 aa) and BrCAX1-2 (36 aa), which was similar to that in AtCAX1.

According to the structural basis of ScVCX1 protein [14], the conserved residues that were essential for CAX function were identified in BrCAX proteins (Figure 4). The residues of S129 (M4), S132 (M4), N299 (M8), H303 (M8), and Q328 (M9) were crucial for the structural maintenance of CAX protein and marked in blue arrow. The residues of E106 (M3) and E302 (M8) were key for Ca^2+^ transport and marked in green arrow, and their interacting residues of G102 (M3), N103 (M3), G298 (M8), and S325 (M9) in purple arrow were also found in an active site. In addition, three interacting residues (in red arrow) of E83 (M2), E230 (acidic helix), and D234 (acidic helix) in another active site were reported to respond to the cellular Ca^2+^ concentration. The identification of these highly conserved residues provides rich information for studying the putative ion transport functions of BrCAX proteins.

### 3.5. Identification of Cis-Acting Elements in the Promoters of BrCAX Genes

**Figure 5:**
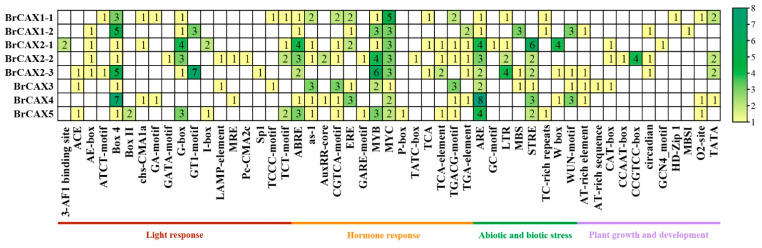
Analysis of cis-acting elements in *BrCAX* promoters. The different colors and numbers on the grid indicate the numbers of different promoter elements.

To understand the regulatory patterns of *BrCAX* family genes, the cis-acting elements in the 2.0 kb region upstream of *BrCAXs* were predicted using PlantCARE online searching. The promotor regions of these *BrCAX* genes contained a variety of cis-acting elements, which were classified into four categories: light response, hormone response, biotic and abiotic stress, and plant growth and development (Figure 5). A list of elements related to hormone responses were identified and suggested that hormones may participate in the regulation of *BrCAX* genes. ABA responsive element (ABRE) and GA responsive element (MYB) were enriched in these *BrCAX* genes, with the exception of *BrCAX3* without ABRE and *BrCAX4* without MYB. Moreover, several elements that were associated with drought stress (MBS), temperature stress (LTR), salinity stress (STRE), and wound stress (WUN-motif) were found in these *BrCAX* genes. In addition, several cis-acting elements, such as light responsive element (Box 4), MeJA responsive element (MYC), and anaerobic induction related element (ARE), were most abundant in all the *BrCAX* genes, indicating that the putative roles of *BrCAXs* may respond to the cis-element-related regulatory processes or stress responses.

### 3.6. Expression Analysis of BrCAX Genes in Different Tissues and under Heat Stress

**Figure 6:**
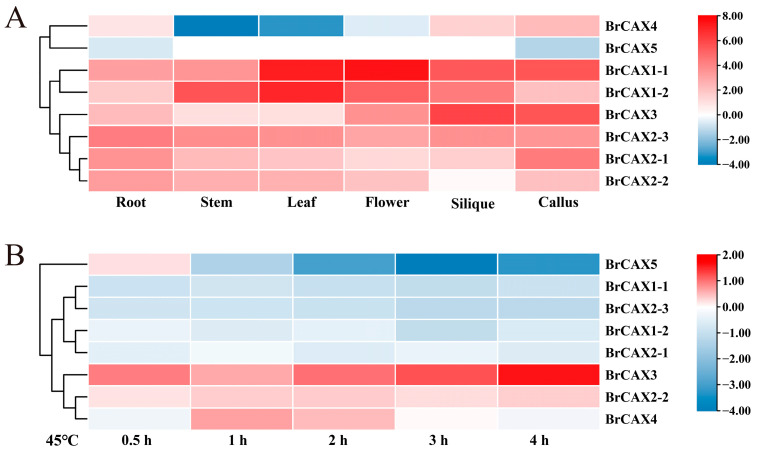
Expression profiles of *BrCAX* genes in different tissues (**A**) and under heat stress (**B**).

Based on the available RNA-Seq data on Chinese cabbage ‘Chiifu’ [38], the tissue-specific expression patterns of *BrCAX* genes in six different tissues (root, stem, leaf, flower, silique, and callus) were analyzed using FPKM values (Table S4). The results showed that the expression levels of eight *BrCAX* genes varied in different tissues (Figure 6A). *BrCAX1-1* and *BrCAX1-2* genes were relatively highly expressed in all the tissues, suggesting the overall involvements and the primarily roles of the two genes during the process of plant development. *BrCAX3* exhibited high expressions in the silique and callus. *BrCAX2-1* exhibited high expressions in the root and callus. The other genes showed low expressions in all the tissues, especially the *BrCAX5* gene with FPKM values < 1 (Table S4).

Furthermore, to investigate the expression changes of *BrCAX* genes in response to 45 °C heat stress, a differential expression pattern of *BrCAX* genes was analyzed using previous microarray data of Chinese cabbage ‘Chiifu’ [39]. The results showed that five genes, such as *BrCAX1-1*, *BrCAX1-2*, *BrCAX2-1*, and *BrCAX2-3*, were down expressed in all the time points under heat stress (Figure 6B; Table S4), but two genes of *BrCAX2-2* and *BrCAX3* were up expressed constantly. The expression of *BrCAX4* exhibited a lowest level at 0.5 h and a highest level at 1 h under stress and then was gradually declined. The expression of *BrCAX5* exhibited a highest level at 0.5 h under stress and then was gradually declined.

### 3.7. Tip-Burn Severity and Ca^2+^ Content Determination of Chinese Cabbage under Ca^2+^ Deficiency Stress

**Figure 7:**
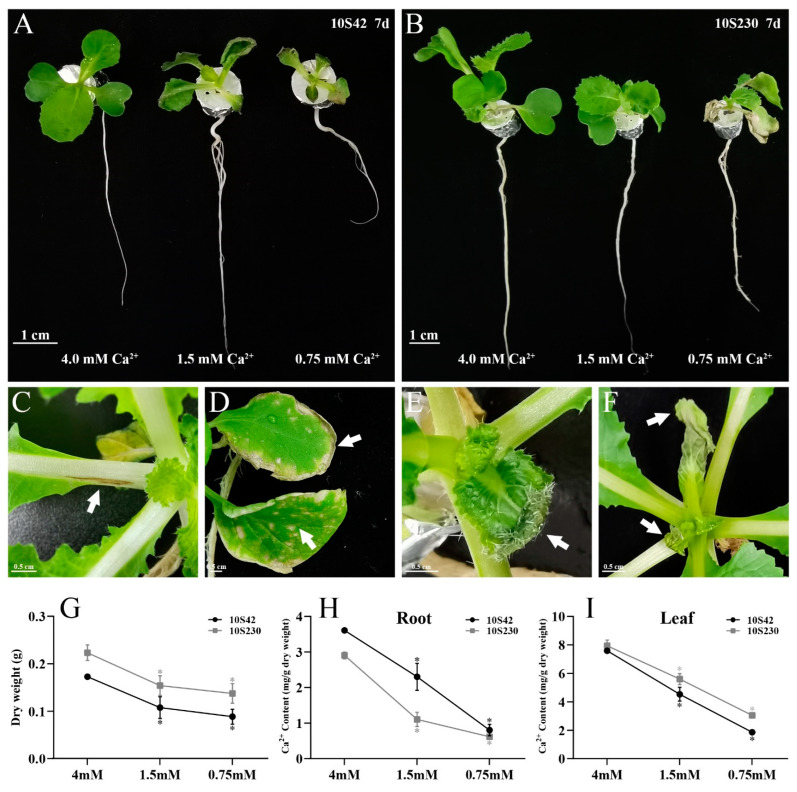
Observation of Ca^2+^ deficiency-induced tip-burn in Chinese cabbage ‘10S42′ and ‘10S230′ lines. (**A**,**B**) The seedlings at 7 d after Ca^2+^ deficiency treatment. (**C**–**F**) The symptoms of tip-burn at 28 d after Ca^2+^ deficiency treatment. White arrows indicated the different symptoms. (**C**) Small black spots in petiole. (**D**) Withered leaf margin and withered spots. (**E**) New leaf curling. (**F**) New leaf shrinking and wilting. (**G**) Statistics of dry weight of seedlings at 28 d after Ca^2+^ deficiency treatment. (**H**,**I**) Total Ca^2+^ content in the root and leaf. Scale bar = 1 cm (**A**,**B**) and 0.5 cm (**C**–**F**). Each bar shows the mean ± SEM of three independent replicates. * *p* < 0.05.

To validate the tip-burn severity of Chinese cabbage ‘10S42′ and ‘10S230′ lines, the seedlings were exposed to Ca^2+^ deficiency treatments using Hoagland solution with different Ca^2+^ concentration. Both the two lines of ‘10S42′ and ‘10S230′ grew normally under 4.0 mM Ca^2+^ condition (CK group). The symptoms of tip-burn were observed in the seedlings under 1.5 and 0.75 mM Ca^2+^ deficiency conditions (1.5 and 0.75 groups) (Figure 7A,B). The typical symptoms, such as small black spots in petiole, withered leaf margin, withered spots, new leaf curling, and new leaf shrinking and wilting (Figure 7C–F), were found at 28 d after treatment. Moreover, the dry weight of seedlings and the total endogenous Ca^2+^ concentration of the root and leaf exhibited a significantly concentration-dependent decrease in the 1.5 and 0.75 groups of both the two lines (Figure 7G–I). Notably, although the Ca^2+^ concentration in the root of susceptible line ‘10S42′ was higher than that in the root of resistant line ‘10S230′ (Figure 7H), the Ca^2+^ concentration in the leaf of susceptible line ‘10S42′ was lower than that in the leaf of resistant line ‘10S230′ (Figure 7I), which may be related to the difference in tip-burn severity between the two lines.

### 3.8. Expression Patterns of BrCAX Genes under Ca^2+^ Deficiency Stress

**Figure 8:**
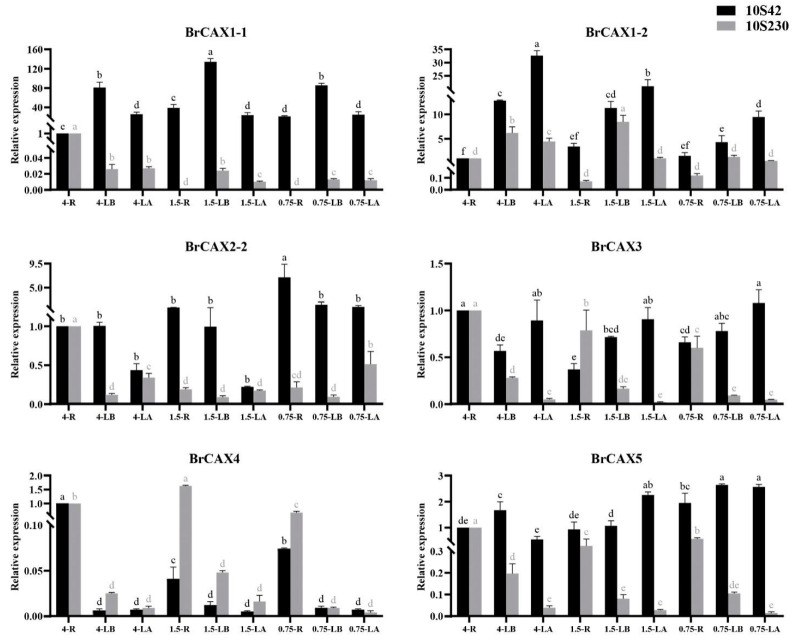
Expression analyses of *BrCAX* genes under Ca^2+^ deficiency stress in Chinese cabbage, where 4, 1.5, and 0.75 indicate the treatments with 4.0, 1.5, and 0.75 mM Ca^2+^ conditions. R, root; LB, leaf base; LA, leaf apex. Each bar shows the mean ± SEM of three independent replicates. The values with different letters indicate significant differences at *p* < 0.05.

To explore the response of *BrCAX* genes to Ca^2+^ deficiency stress, the expression levels of *BrCAX* genes were analyzed in the root, leaf base, and leaf apex from the two lines after Ca^2+^ deficiency treatments. The results showed that these *BrCAX* genes were differentially expressed in different tissues and under different Ca^2+^ deficiency treatments (Figure 8), with the exception that *BrCAX2-1* and *BrCAX2-3* genes were not expressed in all the samples. The expression level of *BrCAX1-1* gene in the three tissues of ‘10S230′ line was decreased in the 1.5 and 0.75 groups compared with that in CK group and was lowest in the root of CK group in ‘10S42′ line and highest in the root of CK group in ‘10S230′ line. Notably, in nearly all the groups, the level of *BrCAX1-1* gene was lower in the leaf apex than leaf base. The expression level of *BrCAX1-2* gene in the leaf base and leaf apex of the 0.75 groups was decreased compared with that in the same tissues of the 4.0 and 1.5 groups both in the two lines. Moreover, the level of *BrCAX1-2* gene was higher in the leaf apex than leaf base in all the groups of ‘10S42′ line, but it was lower in the leaf apex than leaf base in all the groups of ‘10S230′ line. Three genes of *BrCAX2-2*, *BrCAX3*, and *BrCAX5* showed highest expressions in the root of CK group of ‘10S230′ line. The level of *BrCAX2-2* gene was lower in the leaf apex than leaf base in all the groups of ‘10S42′ line. The levels of both *BrCAX3* and *BrCAX5* genes were lower in the leaf apex than leaf base in all the groups of ‘10S230′ line. The *BrCAX4* gene showed high expression levels in the root of all the groups of both ‘10S42′ and ‘10S230′ lines. In addition, the level of *BrCAX4* gene was lower in the leaf apex than leaf base in all the groups except for the CK group of ‘10S42′ line. The expression profiles of *BrCAX* genes provide an important reference for further investigation of *BrCAX* functions.

### 3.9. Prediction of Interaction Network of BrCAX Proteins

**Figure 9:**
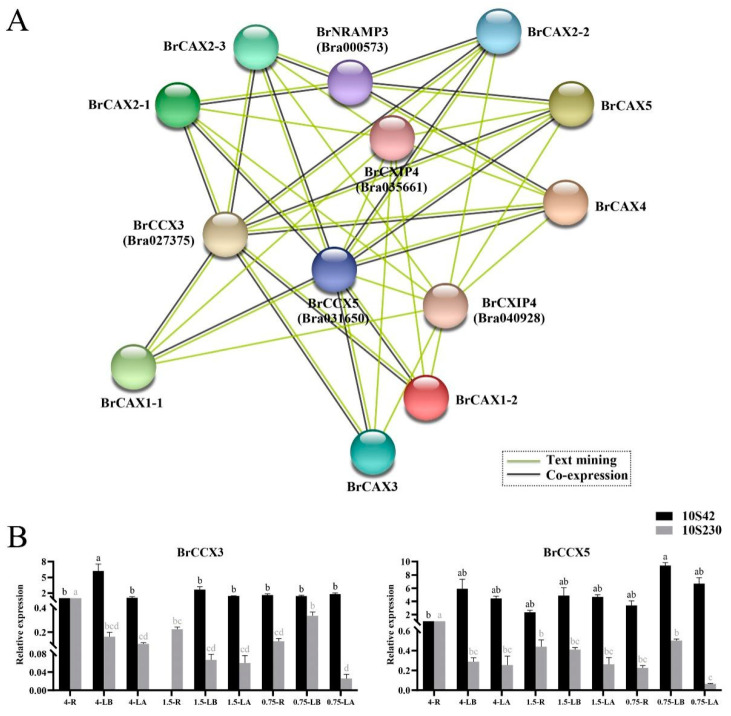
The predicted interaction network among BrCAX proteins (**A**) and expression analysis of *BrCCX* genes under Ca^2+^ deficiency stress (**B**). Each bar shows the mean ± SEM of three independent replicates. The values with different letters indicate significant differences at *p* < 0.05.

To well understand the functions of BrCAX proteins in Ca^2+^-mediated signaling pathways, a putative protein interaction network was predicted using an online STRING server against the genomic information of *B. rapa* (Figure 9A). Among eight BrCAX proteins, no potential relationships were found in the network. Nevertheless, five interacting proteins, including Bra027375 (BrCCX3), Bra031650 (BrCCX5), Bra000573 (BrNRAMP3), Bra035661 (BrCXIP4), and Bra040928 (BrCXIP4), were identified and exhibited putative interaction relationships with BrCAX proteins. In the interaction network, BrCCX3 and BrCCX5, the members of CCX subfamily proteins, showed the parallel relationships and co-expression patterns with all the BrCAXs, suggesting that BrCAX proteins may play similar biological roles to both the two BrCCX proteins. The BrNRAMP3 protein encoding a member of Nramp2 metal transporter family showed the co-expression patterns with BraCAX2-1, BraCAX2-2, BraCAX2-3, BraCAX4, and BraCAX5. In addition, two BrCXIP4 proteins, the known CAX-interacting protein, showed the potential relationships with all the BrCAXs.

The CCX3 and CCX5, also belonging to CaCA superfamily, were reported to mediate the transmembrane transport of various cations [48,49]. Furthermore, expression patterns of *BrCCX3* and *BrCCX5* genes under Ca^2+^ deficiency stress were analyzed in different tissues of Chinese cabbage ‘10S42′ and ‘10S230′ lines. The results showed that *BrCCX3* and *BrCCX5* genes have almost similar expression patterns (Figure 9B). Both *BrCCX3* and *BrCCX5* genes exhibited the lowest expressions in the root of CK group of ‘10S42′ line and highest expressions in the root of CK group of ‘10S230′ line, suggesting that their expression changes may be related to the response of Ca^2+^ deficiency stress. Moreover, the expressions of *BrCCX3* and *BrCCX5* genes were lower in the leaf apex than leaf base in all the groups except for the 0.75 groups of ‘10S42′ line, which was in agreement with the expression profiles of *BrCAX* genes. Expression characteristics of *BrCCX3* and *BrCCX5* genes could contribute to validate the co-expression relationships between *BrCCX* and *BrCAX* genes.

### 3.10. Validation of Ca^2+^ Transport Capacity of BrCAX1-1 and BrCAX1-2 Genes in Yeast and Chinese Cabbage Cotyledons

**Figure 10:**
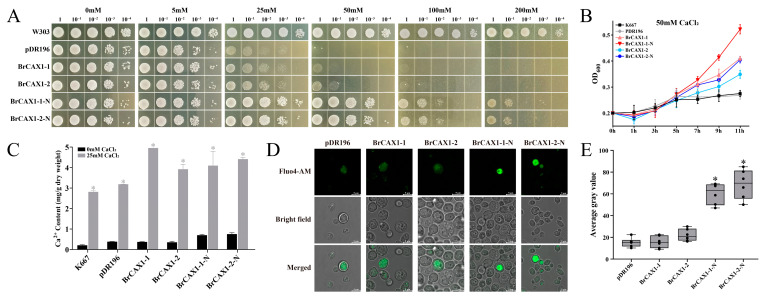
Characterization of Ca^2+^ transport activity of *BrCAX1-1* and *BrCAX1-2* genes in yeast. (**A**) The growth in yeast cells of 10-fold serial dilutions under different concentrations of CaCl_2_ treatment. (**B**) The growth curve in yeast under 50 mM CaCl_2_ treatment. (**C**) Total Ca^2+^ content of yeast cells after 25 mM CaCl_2_ treatment. (**D**) Fluorescence microscopy of yeast cells labelled with Ca^2+^ fluorescent probe Fluo4-AM. Scale bar = 5 μm. (**E**) The average grey values of green fluorescence. Each bar shows the mean ± SEM of three independent replicates. * *p* < 0.05.

Overexpression of *BrCAX1-1* and *BrCAX1-2* genes in a yeast mutant k667 (Ca^2+^ sensitive strain) was performed to investigate their Ca^2+^ transport capacity. Moreover, the N-terminal autoinhibitory region that affect Ca^2+^ transport was removed from their full-length gene sequences, and the truncated *BrCAX1-1-N* and *BrCAX1-2-N* were then transferred to k667 strain. As expected, under high Ca^2+^ treatment, *BrCAX1-1* and *BrCAX1-2* did not promote the growth in yeast, while the *BrCAX1-1-N* and *BrCAX1-2-N* significantly recovered the growth in yeast (Figure 10A). The growth curves also exhibited the increased yeast tolerance to high Ca^2+^ stress in *BrCAX1-1-N* and *BrCAX1-2-N* overexpressed yeast (Figure 10B). Meanwhile, the total Ca^2+^ content was significantly increased in all the yeast cells after high Ca^2+^ treatment (Figure 10C). Furthermore, fluorescence staining showed that strong Ca^2+^ fluorescent signaling in the vacuole was observed in *BrCAX1-1-N* and *BrCAX1-2-N* overexpressed yeast cells (Figure 10D,E), indicating that more Ca^2+^ was accumulated in the vacuole. These results suggest that the N-terminal truncated *BrCAX1-1-N* and *BrCAX1-2-N* have the function of Ca^2+^ transport and promote the accumulation of Ca^2+^ in the vacuole.

**Figure 11:**
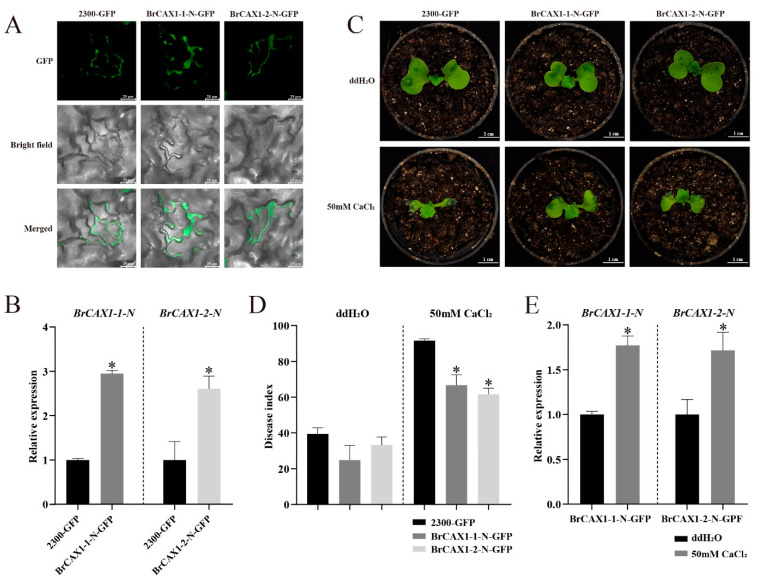
Transformation of *BrCAX1-1-N* and *BrCAX1-2-N* genes in Chinese cabbage cotyledons. (**A**) Observation of GFP fluorescence. (**B**) Gene expression validation in overexpressed cotyledons. (**C**) Symptom of cotyledon necrosis after spraying CaCl_2_ solutions. (**D**) Statistics of disease index of cotyledon necrosis. (**E**) Expression analysis of *BrCAX1-1-N* and *BrCAX1-2-N* genes after spraying CaCl_2_ solutions. As shown, 2300-GFP indicates the control group by expressing the empty vector. BrCAX1-1-N-GFP and BrCAX1-2-N-GFP indicate the overexpression of *BrCAX1-1* and *BrCAX1-2* genes. Scale bar = 25 μm (**A**) and 1 cm (**C**). Each bar shows the mean ± SEM of multiple replicates. * *p* < 0.05.

Furthermore, *BrCAX1-1-N* and *BrCAX1-2-N* genes were overexpressed in Chinese cabbage cotyledons by transient transformation, according to the previous reports [35,44]. The obvious GFP fluorescence and increased gene expression were observed in the cotyledons after infiltrating (Figure 11A,B), indicating the successful transformation of *BrCAX1-1-N* and *BrCAX1-2-N* genes in Chinese cabbage cotyledons. After spraying 50 mM CaCl_2_ solutions, the *BrCAX1-1-N* and *BrCAX1-2-N* overexpressed cotyledons exhibited significantly decrease in disease index of cotyledon necrosis (Figure 11C,D). In addition, the expression levels of *BrCAX1-1* and *BrCAX1-2* genes were significantly increased after spraying CaCl_2_ solutions (Figure 11E), indicating that the overexpression of *BrCAX1-1-N* and *BrCAX1-2-N* promoted Ca^2+^ transport and improved tolerance to environmental Ca^2+^ stress.

### 3.11. Suppression Expressions of BrCAX1-1 and BrCAX1-2 Genes Reduced the Cytosolic Ca^2+^ Levels in the Root Tips of Chinese Cabbage

**Figure 12:**
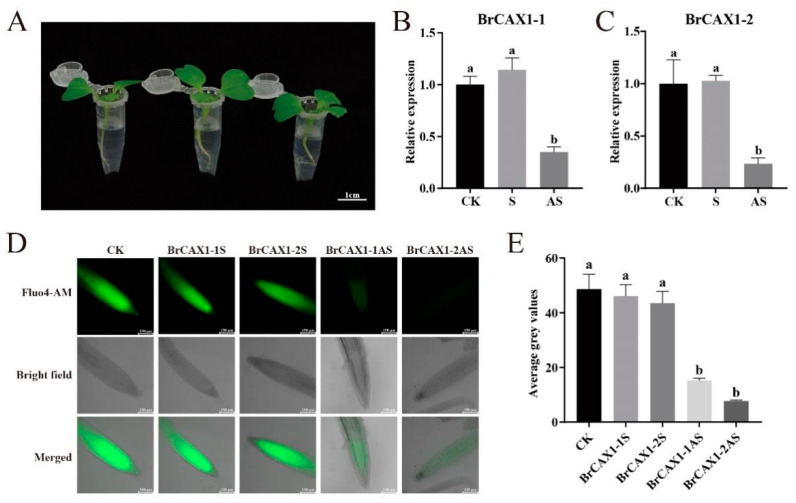
Suppression of *BrCAX1-1* and *BrCAX1-2* gene expression in Chinese cabbage by AS-ODN treatment. (**A**) The seedlings were subjected to ODN treatment. (**B**,**C**) Gene expression validation of *BrCAX1-1* and *BrCAX1-2* after AS-ODN treatment. (**D**) Observation of Ca^2+^ fluorescence in the root tips. (**E**) The average grey values of Ca^2+^ fluorescence in the root tips. Scale bar = 1 cm (**A**) and 150 μm (**D**). Each bar shows the mean ± SEM of multiple replicates. The values with different letters indicate significant differences at *p* < 0.05.

To further validate the functions of *BrCAX1-1* and *BrCAX1-2* genes in Ca^2+^ transport capacity, suppression expression of *BrCAX1-1* and *BrCAX1-2* was performed in Chinese cabbage ‘10S42′ seedlings by AS-ODN treatment (Figure 12A). The specific AS-ODN treated seedlings exhibited the significantly suppressed expression of *BrCAX1-1* and *BrCAX1-2* genes (Figure 12B,C). Observation of Ca^2+^ fluorescence showed that suppression of *BrCAX1-1* and *BrCAX1-2* genes exhibited significantly weaker fluorescent signaling in the root tips (Figure 12D,E), which suggested the decreased cytosolic Ca^2+^ levels. These results further indicate that *BrCAX1-1* and *BrCAX1-2* genes play critical roles in mediating Ca^2+^ transport.

## 4. Discussion

Plant Ca^2+^ deficiency usually causes a physiological disorder, named BER or tip-burn, in rapidly growing tissues of many horticultural crops and leads to destructive damages and significant yield losses for crop production [36]. Generally, the supplement of exogenous Ca^2+^ can reduce the incidence of Ca^2+^ deficiency disorder [50]. Nevertheless, several studies have observed that the increase in total Ca^2+^ concentration of tissues is still accompanied by the Ca^2+^ deficiency-induced symptoms [30,51,52], which are caused by cellular localized Ca^2+^ deficiency actually. The abnormal Ca^2+^ partitioning and distribution at cellular level are crucial factors for triggering Ca^2+^ deficiency disorder, which is concerned with a complex physiological regulatory process and the involvement of Ca^2+^ transporters [30]. CAX proteins are widespread transporter proteins in plant species and mediate the transmembrane movement of Ca^2+^ responding to diverse environmental conditions and stresses [5]. Although the *CAX* subfamily members have been extensively characterized in multiple species, few studies focus on the comprehensive identification and functional exploration of *BrCAX* genes in Chinese cabbage. Therefore, in this study, the systematical characterization and expression analysis of *BrCAX* family genes were performed to further understand the putative functions of BrCAX proteins in Chinese cabbage.

The expansion of a gene family is always accompanied by the tandem and segmental duplication events in species [53,54]. It is known that the *Brassica* species have undergone a whole genome triplication (WGT) event in the long evolutionary history [55,56], which provides the possibility to generate more members of gene family with divergent functions as well as duplicated genes. In this study, eight candidate sequences of *BrCAX* gene family were identified from the genome of *B. rapa*. Compared with *A. thaliana* and *A. lyrata*, more members of *CAX* gene family also exist in other *Brassica* species, such as *B. napus* and *B. oleracea*, which confirmed the widespread existence of duplicated genes throughout the *Brassica* genus and genome duplications. Additionally, the number of *CAX* family members varied among different species that may be due to gene loss event. The whole genome duplication is usually followed by the substantial gene loss, which is regarded as the major source of adaptive functional novelty in plants [57,58]. The widespread members of *CAX* subfamily display a characteristic of phylogenetic diversity within many species. Phylogenetic analysis of our study showed that the CAX proteins from *Brassica* species shared phylogenetic characteristics with five distinct gene subgroups. Moreover, analysis of evolutionary selective pressure showed the various values of *Ka*, *Ks*, and *Ka*/*Ks* ratio among the pairwise comparisons of CAX homologs. The variation of *Ks* values representing the divergence time suggested that these CAX genes diverged concurrently with the *Brassica*-specific WGT event. In addition, a higher value of *Ka*/*Ks* ratio was reported to indicate the genes that may be more likely to evolve new functions during the long evolutionary time [54,57]. In the present study, the CAX1 and CAX2 homologs displayed the higher *Ks* values and lower *Ka*/*Ks* values, indicating the ancient evolutionary division but high evolutionary conservation in CAX1 and CAX2 genes.

Although CAX family has multimember divergence, the CAX homologous proteins commonly share high structural conservation [10,14]. Insights into the conserved functions of CAXs are primarily supported by protein structural characteristics, which are essential for their intrinsic roles in cation transport and homeostasis [15,16]. CAX proteins have evolved the conserved structural components with 11 TM helices and two *α*-repeat regions that include conserved residues for cation binding [14]. As expected, our studies found the high conservation of BrCAX proteins in core regions and residues through multiple sequence alignments against AtCAX and ScVCX1 proteins. The parallel sequence features between BrCAXs and known CAX proteins are responsible for further structural and functional analysis of BrCAX proteins. In this study, the ScVCX1 protein structure was used as a template for protein homology modelling to predict the crystal structures of BrCAX proteins. Structural prediction showed a high similarity between BrCAX and ScVCX1 structures, providing a detailed comprehending of structure-function relationships of BrCAX proteins. The GNxxE signature which is termed *α*-repeat and reported to be opposite in topology [14], contained two inherent residues of E106 and E302 among BrCAXs, which may be important for CAX proteins performing Ca^2+^ binding and transport functions [43]. All the BrCAXs displaying two conserved *α*-repeat regions in an active site may determine their putative ion transport functions. The crystal structure of ScVCX1 shows an acidic helix containing two central residues of Glu and Asp in another active site, and the acidic helix region underneath *α*-repeat region locates on the cytosolic side with parallel orientation to membrane [14]. Among BrCAX proteins, the similar 3D structures with ScVCX1 and the conserved residues of E230 and D234 in acidic helix region were identified, suggesting that BrCAXs may be offered vital roles in specific Ca^2+^ binding. Previous studies have reported that plant CAXs seemed to maintain the structural basis with implications for functional activity in Ca^2+^ homeostasis and signal transduction [59]. In this study, the predicted two active sites in the 3D structures of BrCAX proteins have a possible to be involved in the regulation of Ca^2+^ signaling.

Extensive research on *CAX* genes focused on their physiological and molecular biological roles in diverse aspects of plant growth and development and in response to external stresses [5,11]. In the current study, tissue-specific expression analysis by RNA-Seq showed that these *BrCAX* genes had various expression levels in different tissues, indicating that these *BrCAXs* may participate in diverse biological processes. *BrCAX1-1* and *BrCAX1-2* genes had higher expression levels in all the tissues than other genes, implying that the two genes may be the most actively expressed gene members. It is well known that CAX1 acts as a central ion transporter and has unique responses to biotic and abiotic stresses [5,22]. Zhang et al. [60] reported that *Arabidopsis cax1* mutant enhanced the resistance against avirulent biotrophic pathogens and the accumulation of defense hormone salicylic acid (SA). Liu et al. [61] reported that *CAX1* gene from *Puccinellia tenuiflora* could complement active Ca^2+^ transporters and confer Ba^2+^ tolerance to yeast. In addition, apple CAX3 protein, a homologous protein to CAX1, was validated to have calcium transport activity in yeast [43]. In this study, a series of cis-acting elements related to hormone responses and biotic/abiotic stresses were identified in the promoter regions of *BrCAX* genes, indicating the putative roles of *BrCAXs* in response to various stress conditions.

Notably, the critical roles of *CAXs* as Ca^2+^ transporters are involved in the process of Ca^2+^ deficiency disorders by controlling Ca^2+^ storage and transport and modulating intracellular Ca^2+^ homeostasis [22,62]. Overexpression of potato *sCAX1* gene showed Ca^2+^ deficiency symptoms in leaf and tuber tissues, which provided insights into the role of *sCAX1* in Ca^2+^ homeostasis [63]. In tomato, transgenic plants overexpressing *sCAX1* and *CAX4* contained significantly more Ca^2+^ [29], and *sCAX1* overexpression induced the mobility of Ca^2+^ to vacuole and a significant increase in the occurrence of BER [30]. In the present study, prediction of subcellular localization showed that BrCAX proteins were localized in the vacuole. Overexpression in yeast showed that the N-terminal truncated *BrCAX1-1-N* and *BrCAX1-2-N* genes improved the tolerance of yeast to high Ca^2+^ stress and promoted the accumulation of Ca^2+^ in the vacuole. The *BrCAX1-1-N* and *BrCAX1-2-N* overexpressed Chinese cabbage cotyledons exhibited lower index of necrosis and indicated high tolerance to environmental Ca^2+^ stress. In addition, suppression of *BrCAX1-1* and *BrCAX1-2* gene expressions reduced the cytosolic Ca^2+^ levels in the root tips of Chinese cabbage. These findings demonstrated that *BrCAX1-1-N* and *BrCAX1-2-N* genes function in Ca^2+^ transmembrane transport.

Furthermore, the transcript levels and expression patterns of *CAX* genes are demonstrated to respond to the change in Ca^2+^ concentration and the occurrence of Ca^2+^ deficiency-induced tip-burn in *Brassica* species [36]. Previous studies in *B. oleracea* have identified several differentially expressed tip-burn related genes [64] and found the differences in *BoCAX2* and *BoCAX5* expressions between tip-burn susceptible and resistant lines [65]. In Chinese cabbage, under Ca^2+^ deficiency condition, the expression levels of two *BrCAX1* homologous genes in tip-burn resistant line were higher than that in susceptible line [52]. In addition, the increases in SA and abscisic acid (ABA) levels were related to tip-burn resistance to Ca^2+^ deficiency stress [52,66]. In this study, Ca^2+^ deficiency treatment induced the occurrence of tip-burn symptoms and a decrease in total Ca^2+^ content in tip-burn susceptible line ‘10S42′ and resistant line ‘10S230′ of Chinese cabbage. The expression changes of *BrCAX* genes exhibited obvious difference between the two lines and were responsive to Ca^2+^ deficiency stresses. Specifically, the expression patterns of *BrCAX1-1* and *BrCAX1-2* genes depended on different Ca^2+^ deficiency treatments, implying the possible participation of *BrCAX1-1* and *BrCAX1-2* in the response process and modulation of tip-burn in Chinese cabbage. Moreover, transformation of Chinese cabbage cotyledons showed that the expressions of *BrCAX1-1* and *BrCAX1-2* genes were significantly increased after spraying CaCl_2_ solutions, further suggesting that *BrCAX* gene expression changes may directly respond to external Ca^2+^ stress. Notably, Ca^2+^ is relatively immobile and not easily circulated to growing parts of the plants, resulting in the differences of Ca^2+^ concentration in different tissues and the occurrence of Ca^2+^ deficiency-induced tip-burn in leafy vegetables, especially in new leaves and leaf apex [34,52]. As expected, we found that the total Ca^2+^content in the leaf of ‘10S42′ was lower than that in ‘10S230′ after Ca^2+^ deficiency treatment, which provides evidence for the occurrence of more serious symptoms of tip-burn in susceptible line ‘10S42′ of Chinese cabbage. Moreover, these *BrCAX* genes showed significantly differential expressions between leaf apex and leaf base, which was consistent with the previous studies that the expressions of tip-burn related genes differed in leaf apex and leaf base [52,65]. In addition, the expressions of two *CAX2* homologous genes, *BrCAX2-1* and *BrCAX2-3*, were not detected in the present study, which was in agreement with the reported studies that CAX2 may do not play a major physiological role in regulating Ca^2+^ homeostasis [23,67]. In general, the overexpression in yeast and Chinese cabbage cotyledons and expression profiles of *BrCAX* genes provide insight into further functional characterizations of *BrCAXs* in Chinese cabbage. 

## 5. Conclusions

In this study, eight genes belonging to five *BrCAX* family members were identified in Chinese cabbage by genome-wide searching. These BrCAX proteins contained conserved Na_Ca_ex domains and shared close phylogenetic relationships with their homologous proteins. Molecular evolution analysis and structural alignment showed the evolutionary conservation of *BrCAX* family genes. Further sequence alignment revealed the highly conserved Ca^2+^ binding residues among BrCAX proteins. Moreover, these *BrCAX* genes exhibited tissue-specific expression patterns and differential expression under Ca^2+^ deficiency treatments. Notably, *BrCAX1-1* and *BrCAX1-2* genes exhibited significantly differential expression in the two different tip-burn sensitive lines of Chinese cabbage under Ca^2+^ deficiency stress. Moreover, the N-terminal truncated *BrCAX1-1* and *BrCAX1-2* genes significantly promoted the accumulation of Ca^2+^ in the vacuole of yeast cells and improved the tolerance of Chinese cabbage cotyledons to environmental high Ca^2+^, which validated the functions of *BrCAX1-1* and *BrCAX1-2* in Ca^2+^ transmembrane transport. These results provide rich information for further functional exploration of *BrCAX* genes in regulating Ca^2+^ deficiency response in *Brassica* species.

## Figures and Tables

**Table 1:** Identification of *BrCAX* family genes in Chinese cabbage.

Gene Name	BRAD	Protein ID	Best Hits	Amino Acid Length	Subcellular Localization
*BrCAX1-1*	Bra017134	XP_009141725.1	AT2G38170	465	vacuole
*BrCAX1-2*	Bra005131	XP_009143416.1	AT2G38170	465	vacuole
*BrCAX2-1*	Bra039385	XP_009124739.1	AT3G13320	472	vacuole
*BrCAX2-2*	Bra034690	XP_009146534.1	AT3G13320	441	vacuole
*BrCAX2-3*	Bra001499	XP_009135296.1	AT3G13320	497	vacuole
*BrCAX3*	Bra012833	XP_033144772.1	AT3G51860	425	vacuole
*BrCAX4*	Bra009640	XP_009122850.1	AT5G01490	521	vacuole
*BrCAX5*	Bra030840	XP_009106834.1	AT1G55730	411	vacuole

## Data Availability

Data are contained within the article or supplementary material.

## Supplementary Materials

The following supporting information can be downloaded at: https://www.mdpi.com/article/10.3390/genes14091810/s1. Figure S1 Prediction and analysis of BrCAX protein 3D structures. (A) Prediction of 3D structure of eight BrCAX proteins. (B) Analysis of the conserved “GNxxE” regions (in pink and wheat) in BrCAX2-1, BrCAX2-2, BrCAX2-3, and BrCAX5 proteins. Table S1 CAX homologous proteins used for phylogenetic analysis. Table S2 Specific primers used in this study. Table S3 Information of *BrCAX* family genes in Chinese cabbage. Table S4 Expression levels of *BrCAX* genes in different tissues and under heat stress.

## Abbreviations

Ca^2+^: Calcium; PM, plasma membrane; TN, tonoplast; ER, endoplasmic reticulum; TM, transmembrane; CAX, Ca^2+^/H^+^ exchanger antiporter; BER, blossom-end rot; Ka, nonsynonymous substitution rate; Ks, synonymous.
