# Supplementary material for: Genome-Wide Identification of BrCAX Genes and Functional Analysis of BrCAX1 Involved in Ca2+ Transport and Ca2+ Deficiency-Induced Tip-Burn in Chinese Cabbage (Brassica rapa L. ssp. pekinensis)
Source: Genes (Basel). 2023 Sep 17;14(9):1810. doi: 10.3390/genes14091810 (PMC10531375; doi:10.3390/genes14091810)
Supplement: Supplementary file 1 [file genes-14-01810-s001.zip › genes-2617718-supplementary.pdf]

Table S1 CAX homologous proteins used for phylogenetic analysis

Gene name	Gene ID	Gene name	Gene ID
AtCAX1	AT2G38170	BolCAX1-1	Bol017767
AtCAX2	AT3G13320	BolCAX1-2	Bol025283
AtCAX3	AT3G51860	BolCAX2-1	Bol015803
AtCAX4	AT5G01490	BolCAX2-2	Bol035523
AtCAX5	AT1G55730	BolCAX2-3	Bol040372
BnaCAX1-1	BnaA04g21850D	BolCAX3	Bol004999
BnaCAX1-2	BnaA05g06490D	BolCAX4	Bol000685
BnaCAX1-3	BnaC04g07160D	BolCAX5-1	Bol013325
BnaCAX1-4	BnaC04g45720D	BolCAX5-2	Bol035229
BnaCAX2-1	BnaA01g29870D	RsaCAX1-1	Rsa10020334
BnaCAX2-2	BnaA03g32760D	RsaCAX1-2	Rsa10038932
BnaCAX2-3	BnaA05g26020D	RsaCAX2	Rsa10034738
BnaCAX2-4	BnaC01g37750D	RsaCAX3	Rsa10018075
BnaCAX2-5	BnaC03g37860D	RsaCAX4	Rsa10008014
BnaCAX2-6	BnaC05g40050D	RsaCAX5-1	Rsa10012211
BnaCAX3-1	BnaC07g32560D	RsaCAX5-2	Rsa10029090
BnaCAX3-2	BnaA03g41510D	AlCAX1	AL4G35190
BnaCAX4-1	BnaCnng03980D	AlCAX2	AL3G25140
BnaCAX4-2	BnaA10g27650D	AlCAX3	AL5G32320
BnaCAX5-1	BnaA08g00350D	AlCAX4	AL6G10580
BnaCAX5-2	BnaC03g70750D	AlCAX5	AL1G63780
BnaCAX5-3	BnaC06g11060D	–	–

Table S2 Specific primers used in this study

Primer	Sequence (5'-3') forward	Sequence (5'-3') reverse
qRT-PCR analysis		
BrCAX1-1	GTAATCCAGAAGGTTCCATA	CCGCAATAAGTTCCGATA
BrCAX1-2	ATCGCTTACATCGCATAT	TACTCGTCATCCTCATCT
BrCAX2-1	TCGGCTAATGAAGTTGAG	AAGGTAGTAACAGGTTGAG
BrCAX2-2	CGCTTATCTCTTCTTCCA	ATCTTCATCTTCACCACAA
BrCAX2-3	GACGAGAAGAAGACGATT	CAATGGTGTAATGCCTATT
BrCAX3	TGCTTATTGCTTACATTGC	AACGGTCACTTCATCATTA
BrCAX4	GTGAGTGACAAGGAAGAA	ATAGTGGAGACAAGGTAGT
BrCAX5	ACTTCTTCATTACACGCATA	CATAGGCAACGAGCATTA
BrCCX3	GGAGTAGCCAAGAGAATG	CAAGAGCAGAGCCAATAG
BrCCX5	TATGCTTGTTGGACTTGG	AATCTCGGAACTCGGAAT
BrEF-1α	GCCAAAGAGGCCATCAGACAA	ATACCAGGCTTGAGCATACCG
Amplification of gene sequence		
BrCAX1-1 (F)	GGGGATCGTGCAGGAACA	TCGTGAAAGACTCAGGGA
BrCAX1-1 (B)	GAGACCTCTGCTGATTTAA	TTAAGATGAGAAAACTCCTTCG
BrCAX1-2 (F)	ATGGCGGGGATCGTTGAA	TCCCTTCGGAGATTAGCA
BrCAX1-2 (B)	AAAGTCGCAGTGGTAAAG	TTAAGATGAGAAAACGCCTTCG
Construction of yeast expression vector		
BrCAX1-1	AGTGGATCCCCCGGGCTGCAGATGGCGGGGATCGTGCAG	GGGCCCCCCCTCGAGGTCGACTTAAGATGAGAAAACTCCTTCGCC
BrCAX1-1-N	AGTGGATCCCCCGGGCTGCAGATGTCTTCTTCTTCGTTGAGGAAGA	GGGCCCCCCCTCGAGGTCGACTTAAGATGAGAAAACTCCTTCGCC
BrCAX1-2	AGTGGATCCCCCGGGCTGCAGATGGCGGGGATCGTTGAA	GGGCCCCCCCTCGAGGTCGACTTAAGATGAGAAAACGCCTTCGC
BrCAX1-2-N	AGTGGATCCCCCGGGCTGCAGATGTCTTCTTCTTCGTTGAGGAAGA	GGGCCCCCCCTCGAGGTCGACTTAAGATGAGAAAACGCCTTCGC
Construction of GFP expression vector		

BrCAX1-1-N	ATTTGGAGAGGACAGGGTACCATGTCTTCTTCTTCGTTGAGGAAGA	AGCTCCTCCTCCTCCTCTAGAAGATGAGAAAACTCCTTCGCCG
BrCAX1-2-N	ATTTGGAGAGGACAGGGTACCATGTCTTCTTCTTCGTTGAGGAAGA	AGCTCCTCCTCCTCCTCTAGAAGATGAGAAAACGCCTTCGCC
AS-ODN treatment		
BrCAX1-1-S	TTTACTCCTAATGGGACTGT	-
BrCAX1-1-AS	ACAGTCCCATTAGGAGTAAA	-
BrCAX1-2-S	CCGATGTGAACTTCTTCTTA	-
BrCAX1-2-AS	TAAGAAGAAGTTCACATCGG	-

Note: F, the front end sequence; B, the back end sequence; N, the N-terminal truncated sequence. The underlines represent the restriction sites.

Table S3 Information of *BrCAX* family genes in Chinese cabbage

Gene name	Nucleotide ID	Protein ID	CDS length	DNA sequence length	Molecular weight	Theoretical pI	Instability index	Aliphatic index	Grand average of hydropathicity	Number of TM region
BrCAX1-1	XM_009143477.3	XP_009141725.1	1398	3478	50327.84	5.50	33.15	122.30	0.589	11
BrCAX1-2	XM_009145168.3	XP_009143416.1	1398	3857	50321.77	5.52	33.23	120.24	0.561	11
BrCAX2-1	XM_009126491.3	XP_009124739.1	1419	2280	51431.24	5.09	40.50	114.64	0.565	11
BrCAX2-2	XM_009148286.3	XP_009146534.1	1326	2167	48326.67	4.86	39.01	114.33	0.586	11
BrCAX2-3	XM_009137048.3	XP_009135296.1	1494	2904	54679.70	5.40	39.32	107.89	0.427	11
BrCAX3	XM_033288881.1	XP_033144772.1	1278	6739	45970.04	5.32	38.65	128.07	0.661	11
BrCAX4	XM_009124602.3	XP_009122850.1	1566	4346	57536.43	9.21	47.59	111.00	0.365	11
BrCAX5	XM_009108586.3	XP_009106834.1	1236	2178	45189.98	5.02	33.86	119.32	0.643	11

Table S4 Expression levels of *BrCAX* genes in different tissues and under heat stress

Gene name	FPKM value								Probe intensity (PI) value under 45 °C stress					
	Root 1	Root 2	Stem	Leaf 1	Leaf 2	Flower	Silique	Callus	0 h	0.5 h	1 h	2 h	3 h	4 h
BrCAX1-1	10.147	7.963	11.561	150.601	145.783	181.616	43.239	44.906	26325.400	14161.200	14991.600	13305.800	12261.800	13807.000
BrCAX1-2	3.877	2.871	47.972	130.664	113.405	36.708	20.376	4.302	2262.780	1761.760	1464.160	1677.450	1038.040	1430.560
BrCAX2-1	12.976	10.905	4.905	3.954	3.776	2.466	3.145	20.266	1197.530	849.109	1016.310	781.599	932.940	780.350
BrCAX2-2	9.997	9.633	6.263	6.366	5.653	3.999	1.133	4.287	471.360	543.554	605.126	609.848	565.135	607.356
BrCAX2-3	21.028	17.325	13.556	13.961	11.886	7.985	12.522	11.133	3962.340	2212.110	2185.230	2086.810	1734.030	1752.040
BrCAX3	4.485	4.996	2.141	2.468	1.625	12.241	66.067	46.238	1673.860	3163.890	2578.230	3390.220	3919.050	5181.600
BrCAX4	1.957	1.739	0.067	0.075	0.141	0.662	2.812	4.962	393.327	318.474	632.359	552.413	406.801	331.258
BrCAX5	0.666	0.579	0.000	0.000	0.000	0.000	0.000	0.414	321.191	376.524	121.941	40.456	20.379	33.164
