# Supplementary figures and images for: Genome-Wide Identification of BrCAX Genes and Functional Analysis of BrCAX1 Involved in Ca2+ Transport and Ca2+ Deficiency-Induced Tip-Burn in Chinese Cabbage (Brassica rapa L. ssp. pekinensis)
Source: Genes (Basel). 2023 Sep 17;14(9):1810. doi: 10.3390/genes14091810 (PMC10531375; doi:10.3390/genes14091810)

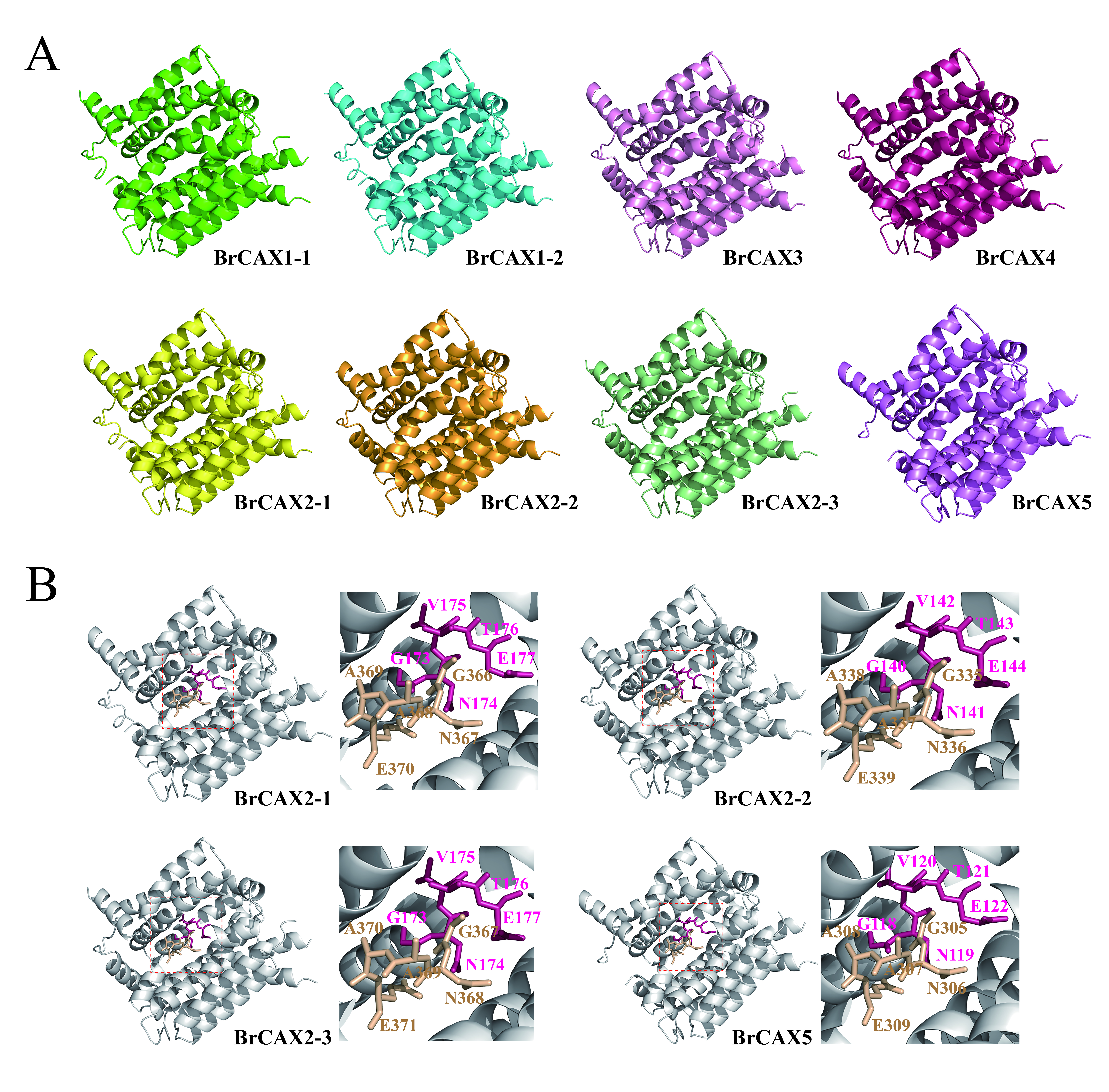

Supplement: Supplementary file 1 [file genes-14-01810-s001.zip › Figure S1(1000dpi 18cm).jpg]
